# Fred W. van Leeuwen (1949–2021)

**DOI:** 10.1186/s13024-021-00444-5

**Published:** 2021-03-31

**Authors:** Bert M. Verheijen, Aaron Ciechanover

**Affiliations:** 1 Leonard Davis School of Gerontology, University of Southern California, Los Angeles, CA 90089 USA; 2 The Rappaport Faculty of Medicine and Research Institute, Technion-Israel Institute of Technology, 31096 Haifa, Israel

Frederik (Fred) Willem van Leeuwen (Amsterdam, 1949), a Dutch neurobiologist, passed away on January 13, 2021 after a period of illness. Fred was well known internationally for his contributions to the fields of neuropeptides and neurodegeneration, and in particular for his breakthrough discovery on frameshift mutants of ubiquitin (Ub) that are present in the neuritic plaques and neurofibrillary tangles that characterize Alzheimer’s disease (AD). This remarkable discovery provided the first compelling mechanistic evidence for the involvement of the Ub-proteasome system (UPS), the major intracellular proteolytic pathway, in AD pathogenesis. The evidence for the involvement of the UPS in neurodegeneration has grown ever since exponentially, and has engulfed literally all neurodegenerative disorders in different mechanisms of protein quality control.

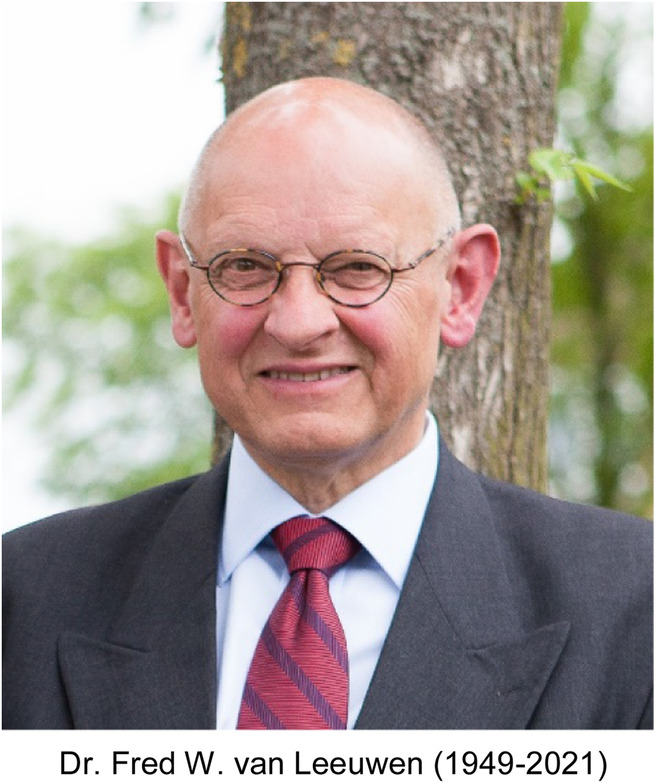



Fred was trained in neurobiology at the Netherlands Institute for Brain Research, part of the Royal Netherlands Academy of Arts and Sciences (KNAW), in Amsterdam (PhD 1980; Thesis: ‘Light and electron microscopical immunocytochemical localization of neuropeptides in the rat brain’, promotor: Prof. Dr. D.F. Swaab, Vrije Universiteit, Amsterdam). There, he conducted research on neuropeptides and the brain hypothalamo-neurohypophyseal system. Of note are his pioneering studies on the extrahypothalamic localization of vasopressin (VP), oxytocin and other neuropeptides, the role of neuropeptide innervation of pituicytes in the release of VP and oxytocin in the neurohypophysis, and the specificity problems of immunocytochemistry. Importantly, he also carried out work on the Brattleboro rat, a natural knockout model for VP. The homozygous Brattleboro rat suffers from central diabetes insipidus due to a single base deletion in the VP peptide hormone precursor. This deletion causes a − 1 frameshift, and results in an abnormal VP precursor product that cannot enter the secretory pathway. Surprisingly, it was found that some solitary neurons in the brain of this knockout mouse display immunoreactivity for VP, and that the number of VP-positive cells in the brain increased progressively with age. Further investigation by Fred and his team revealed that this revertant phenotype was apparently caused by additional transcriptional dinucleotide deletions (ΔGA) occurring on simple repeat motifs (GAGAG) present in the VP gene. These dinucleotide deletions, generated by misreading of the genetic code, resulted in restoration of the wild-type (WT) reading frame and synthesis of a functional VP molecule.

Based on the observations in the Brattleboro rat, it was hypothesized that molecular misreading events of transcripts (there are no mutations in the coding gene) may also occur on dinucleotide repeat motifs present in other genes, and that such events might even contribute to age-related neurodegenerative disease. Interestingly, Fred’s team demonstrated that similar + 1 frameshift mutants could indeed be detected in the brains of patients with early onset AD and Down syndrome (the latter also exhibit AD-like brain pathology when middle-aged). In particular, a frameshift mutant of Ub, dubbed UBB^+ 1^, was shown to accumulate as a neuropathological hallmark in these diseases. The frameshift results in addition of a 19 amino acids tail to the molecule which alters the ‘canonical’ C-terminal glycine residue of the molecule. In the WT molecule, this moiety is activated by the Ub-activating enzyme, E1, and is then covalently conjugated to the target proteins (and to previously conjugated Ub moieties) via an isopeptide bond (to the ε-NH_2_ amino group of an internal Lys residue), signaling them for proteasomal degradation. The alteration in the C-terminal residue abrogates the conjugating ability of Ub. It was established that UBB^+ 1^ is a dose-dependent inhibitor of the UPS, and that expression of UBB^+ 1^ in experimental model systems results in phenotypes that are compatible with neurodegeneration. UBB^+ 1^ was also detected in protein aggregates in an array of other neurodegenerative diseases, specifically in tauopathies (e.g., Pick’s disease), and polyglutamine repeat disorders (e.g., Huntington’s disease). It is plausible that UBB^+ 1^ contributes to disease progression by influencing cellular quality control pathways (as while it cannot conjugate to target proteins signaling them for degradation, it can deplete intracellular Ub pools by being conjugated by WT Ub), and by modifying the aggregation and toxicity of other disease-associated proteins.

While the exact molecular origins of the + 1 frameshift mutants have remained elusive and the biological consequences of UBB^+ 1^ expression remain to be explored in more detail, the findings suggested a novel mechanism of neurodegeneration that depends on the UPS. Extended Ub mutants have also been used as tools to selectively probe the Ub landscape in cells.

Fred was recruited in 2007 to the Maastricht University Department of Psychiatry and Neuropsychology, part of the School for Mental Health and Neuroscience at the Faculty of Health, Medicine and Life Sciences where he remained until his retirement in 2016. After his retirement, he remained active as a scientist and continued to publish on the UBB^+ 1^ mutant.

All of us who had the honor of working with Fred remember him as an enthusiastic and engaged scientist and a friendly, loyal and supportive colleague. This was exemplified by his generous sharing of knowledge and resources. He invested a lot of his time in teaching and mentoring, and in organizing events that aimed to stimulate contacts between neuroscientists. For example, he organized international practical courses on Immunocytochemistry (EMBO) and on Molecular Neuroanatomy (ETP/ENA/IBRO), and was an initiator and driving force behind the Endo-Neuro-Psycho (ENP) meetings in the Netherlands, currently known as the Dutch Neuroscience Meetings. He remained fully devoted to his family and scientific work until his death. Fred and his frameshifting thinking will be deeply missed by his colleagues, trainees and friends.

Fred is survived by his wife Elly, his son Roelof and his daughters Sara and Hilde, and their children.

*We thank Elly van Leeuwen, Gerard Boer and Dick Swaab for helpful suggestions.*

